# Circadian cycle and neuroinflammation

**DOI:** 10.1515/biol-2022-0712

**Published:** 2023-10-17

**Authors:** Xinzi Xu, Junli Wang, Guohua Chen

**Affiliations:** Department of Neurology, Wuhan No. 1 Hospital, Wuhan 430022, China; College of Clinical Chinese Medicine, Hubei University of Chinese Medicine, Wuhan 430065, China

**Keywords:** circadian rhythm, neuroinflammation, microglia, astrocyte

## Abstract

Circadian cycle is a fundamental characteristic of life formed in the long-term evolution of organisms and plays an important role in maintaining the proliferation, migration, and activation of immune cells. Studies have shown that circadian rhythm disorders affect the occurrence and development of neuroinflammation by inducing glial cell activation and peripheral immune responses. In this article, we briefly described the research progress of neuroinflammation and circadian rhythm in recent years and explored the effects and possible mechanism of circadian rhythmicity on microglia, astrocytes, and peripheral immune function.

## 1 Introduction

Neuroinflammation refers to a wide range of inflammatory responses that occur in the central nervous system (CNS), characterized by glial cell activation, production, and release of inflammatory mediators, and recruitment of peripheral immune cells [1]. Neuroinflammation is an important physiological response of the brain to defend against infections and injuries, promote tissue repair, and clear cellular debris and pathogens [2]. However, a large number of pro-inflammatory mediators could be released under excessive inflammatory stimulation, which leads to blood-brain barrier (BBB) leakage and neurotoxic damage, thus participating in the pathological processes of various neurological and psychiatric disorders [3].

The circadian cycle is the regular biological activity variation with the day-night cycle as its period that organisms have developed through long-term evolution. Studies have found that the disruption of the circadian cycle is closely related to the immune function and inflammatory response of the body [4]. Almost all immune cells in humans and animals (such as microglia, neutrophils, monocytes, and lymphocytes) could express clock genes [5,6] and are involved in regulating the maturation and activation of immune cells [7,8]. Therefore, the disruption of the circadian cycle may be an important cause of mediating central and peripheral inflammatory responses. We will review the research progress in recent years on the disruption of the circadian cycle and its regulation of microglia, astrocytes, and peripheral immune cells involved in neuroinflammation.

## 2 Circadian cycle and clock genes

The circadian cycle is a characteristic biological process observed in organisms at both physiological and behavioral levels. For example, animal feeding, activity, sleep-wake cycles [9], as well as fluctuations in heart rate, blood pressure, body temperature, and some hormone levels all exhibit rhythmicity with a period of approximately 24 h [10]. The circadian cycle system consists of three parts: input, central oscillator, and output. The input pathway primarily refers to the components, like the retina, which can perceive changes in external environmental signals such as natural light, temperature, and feeding. These components transmit the changes in signals to the biological clock. The output pathway refers to the various biological rhythms regulated by the biological clock, oscillating near a 24 h cycle and involving various crucial physiological and biochemical processes in the human body. In humans and mammals, the central oscillator is mainly located in the suprachiasmatic nucleus (SCN) of the hypothalamus. As the pacemaker of the circadian cycle (also known as the central clock), it can sense external environmental information conveyed by entrainment factors (such as the light–dark signal projection received by the retinohypothalamic nerve tracts), and then transmit the signal to peripheral clocks through changes in autonomic nervous excitability and neuroendocrine activity, adjusting its own phase to adapt the body to the external environment.

At the molecular level, the circadian cycle is mainly controlled by the transcription-translation negative feedback loop composed of core components of the biological clock. The positive elements mainly include the Clock and Bmal1 genes. As transcription factors, Clock and Bmal1 form a heterodimer through the PAS (PER–ARNT–SIM) domain. This heterodimer binds to the E-box (CACGTC) sequence in the promoter region of downstream genes, driving the transcription of target genes such as Per and Cry. When the expression of Per and Cry increases, they can translocate to the cell nucleus as negative elements and feedback inhibit the activity of Clock–Bmal1. Also, the Clock–Bmal1 heterodimer can activate clock-controlled genes Rev-erb and ROR transcription, which competitively bind to the RORE site on the Bmal1 promoter, thereby influencing the expression of Bmal1 [11]. In addition, genes such as E4BP4, DBP, and NFIL3 can also assist in regulating the expression of the negative feedback loop, jointly maintaining the approximate 24 h periodic oscillation of the biological clock.

Natural light is the most important zeitgeber that acts on organisms, promoting the synchronization of their circadian cycle with periodic environmental changes. Studies have shown that factors such as urban light pollution, nocturne jobs, and jet lag, which cause light signals to be out of sync with the natural light cycle, can suppress melatonin secretion and lead to a disrupted circadian cycle, with reduced amplitude and/or delayed phase. In addition, daily activities such as eating, socializing, and exercise can also affect the dynamic changes of the circadian cycle by influencing the neuroendocrine system, including the secretion of glucocorticoids to achieve synchronization of peripheral clocks [12,13].

## 3 Circadian cycle disturbance and neuroinflammation

In 1960, Halberg et al. first discovered that the mortality rate of mice induced by lipopolysaccharide changed in a time-dependent manner. A dose of endotoxin which is compatible with the survival of most animals when given during the middle of the daily dark period is highly lethal when it is given 8–12 h earlier or later, suggesting that the immune system may have a circadian cycle [14]. Subsequent studies confirmed that the number of circulating white blood cells in the human and animal body shows regular fluctuations within 24 h. For nocturnal mice, the peak occurs mostly around dusk, while for humans it is mostly around 8 am [15]. Further research found that the proliferation, migration, and activation of immune cells are also influenced by the circadian cycle and the expression of clock genes [16], indicating a close association between the circadian cycle and immune inflammation.

### 3.1 Circadian cycle and neuroinflammatory factors

In recent years, studies have suggested that the inflammatory response in the CNS is also regulated by the circadian cycle. Animal models exposed to night-shift work or night light showed significantly increased levels of activated microglia and proinflammatory cytokines in brain tissue, affecting the recovery of neural function in a focal brain ischemia model and inducing anxiety and depressive behavior in mice [17,18]. Sleep deprivation can activate NF-κB and increase the release of inflammatory factors such as IL-1β and TNF-α in the hippocampus, leading to neuronal damage [19]. IL-1β and TNF-α, as important cell factors that mediate inflammatory damage, are believed to be closely related to the regulation of the sleep–wake cycle and sleep phase in the body. Studies have shown that IL-1β and TNF-α mRNA levels in the brain tissue of experimental animals exhibit significant circadian rhythm changes that are consistent with changes in the sleep–wake cycle. Inhibiting the expression of IL-1β and TNF-α can reduce the spontaneous non-rapid eye movement sleep in experimental animals [20,21,22], confirming that pro-inflammatory cytokines may be important factors in mediating the interaction between circadian cycle and neuroimmune function.

### 3.2 Circadian cycle and microglia

Microglia are the most common resident immune cells in the CNS. Lineage tracing studies have shown that microglia originate from precursor cells in the yolk sac erythromyeloid lineage and migrate to the neural tube during early embryonic development. They then settle in the brain parenchyma and maintain their numbers through continuous self-renewal [23]. Microglia exhibit high plasticity and heterogeneity. In the adult brain, microglia in a steady state exhibit a small, highly branched morphology (M0 phenotype) and dynamically sense changes in the microenvironment. They interact extensively with neurons, astrocytes, and oligodendrocytes, among others, to perform important physiological functions such as immune surveillance, synaptic remodeling, and clearance of cellular debris [24,25,26]. Once exposed to abnormal conditions, microglia can be rapidly activated into an amoeboid shape and transform into multiple functional phenotypes in response to various stimuli, thereby enhancing phagocytic activity and regulating inflammatory responses [27]. Receptors associated with phagocytic function include chemokine CX3C receptor 1, triggering receptor expressed on myeloid cells 2, purinergic receptor P2Y12, complement component receptors, and integrin αM subunits, among others [28]. In the classical activation pathway, microglia can be activated to the M1 proinflammatory phenotype by stimuli such as damage or pathogens such as lipopolysaccharide, interferon-γ, and β-amyloid protein. This leads to the release of large amounts of proinflammatory factors such as TNF-α, IL-18, IL-1β, and nitrogen-containing substances and reactive oxygen species, triggering antigen presentation-mediated adaptive immunity [29]. Microglia also exhibit an alternative activation M2 phenotype, which mainly expresses anti-inflammatory cytokines and growth factors such as IL-4, IL-10, and TGF-β. This plays an important role in suppressing inflammatory responses, protecting and repairing neurons, among other functions [30].

In recent years, transcriptomic analysis has revealed functional heterogeneity among subpopulations of microglia [31]. Besides external environmental stimuli, the phenotype of microglia is tightly regulated to some extent by their intrinsic characteristics [32]. It is believed that the endogenous circadian system is one of the important factors influencing microglial activation and phenotype transformation [8]. In 2011, Nakazato et al. first demonstrated the expression of clock genes, such as Per1 and Per2, in primary cultured microglia and BV2 cells [33]. Subsequent studies also confirmed the periodic oscillation of endogenous clock genes in microglia at the level of *in vitro* and *ex vivo* isolation and purification. Among them, the secretion peak of Per1 and Per2 occurs at Zeitgeber time (ZT) 14, which is 2 h into the dark phase, while the peak of Rev-erb appears at ZT18 [5]. It is worth noting that different clock gene expressions can mediate structural and functional changes in microglia, leading them to follow the circadian cycle consistent with the central clock. Studies have shown that compared to the sleep phase (i.e., light phase), microglial processes in the wakefulness phase of the mouse cortex are longer and the number of branches increases [34]. P2Y12 receptor, as a microglial surface-specific protein, is an important signaling molecule that drives its directional movement and cell tropism [35]. During wakefulness, the secretion of cathepsin S by microglia can mediate changes in cell morphology and increase process length by activating its own P2Y12 receptor [34]. On the other hand, the circadian cycle is also closely related to the phagocytic function and immune activity of microglia. Research shows that under conditions of light exposure, the inflammatory activity of rat microglia is enhanced, with a significant increase in the production and release of pro-inflammatory cytokines TNF-α, IL-1β, and IL-6. At this time, the phagocytic function of microglia is weakened, and the expression of surface activation marker protein CD11b, complement pathway, and milk fat globule epidermal growth factor 8 (MFG-E8) are inhibited [36]. MFG-E8 acts as a soluble bridging molecule, which binds to microglia integrin subunit αγβ3 or αγβ5 to induce its phagocytic function. Bmal1, as one of the core clock genes, is believed to be associated with rhythmic changes in microglial function. Wang et al. [37] found that when Bmal1 gene was knocked down in mice and BV2 cell models exposed to LPS, it could lead to downregulation of pro-inflammatory cytokine expression, upregulation of antioxidant and anti-inflammatory gene expression, and the mechanism of which requires further investigation. Clock-controlled gene Rev-erb also plays an important role in the molecular clock and neuroinflammatory regulation of microglia. Wolff et al. observed that after intervening with the primary microglia cells using the Rev-erb gene activator SR9011, SR9011 interferes with the rhythmic secretion of cells, participates in inhibiting their inflammatory activity and phagocytic function, and downregulates the mitochondrial respiration and ATP production of microglial cells [38]. In addition, research shows that the negative feedback transcription factor E4bp4 can also downregulate the activation of microglia by directly binding to the D-box element in the promoter region to inhibit the MAPK/ERK signaling pathway [39].

### 3.3 Circadian cycle and astrocytes

Astrocytes are the largest type of glial cells in the CNS, with multiple elongated branches that fill the CNS. They form extensive connections with neurons, oligodendrocytes, and microglia, and participate in important processes such as synapse formation, regulation of neuronal activity, and maintenance of the integrity of the BBB in physiological conditions [40]. However, in pathological situations such as trauma, infection, and ischemia, astrocytes rapidly respond by forming reactive astrocytes (RAS) characterized by hypertrophy, proliferation, and molecular remodeling [41]. Currently, it is generally believed that RAS induced by inflammatory and ischemic stimuli have significant differences in gene expression and are respectively named classical astrocytes (A1 type) and selective astrocytes (A2 type) [42]. A1-type astrocytes are the main source of the synaptic destructive classical complement cascade components (C3 and C4B), and can release neurotoxins such as long-chain saturated lipids, causing neuronal and oligodendrocyte death. A2-type astrocytes can release various neurotrophic factors such as vascular endothelial growth factor and have neuroprotective effects [43]. Therefore, astrocytes also play an important role in neuroinflammatory reactions.

Traditional views hold that the circadian cycle is controlled by the electrical activity coupling of different neuron subgroups within the SCN. However, recent studies have found that astrocytes are also an indispensable part of the regulation of the SCN circadian cycle [44]. On the one hand, astrocytes can influence neuronal activity by regulating extracellular levels of gamma-aminobutyric acid (GABA) and glutamate, thereby mediating the regulation of the SCN circadian cycle [45,46]. When glutamate release from astrocytes is inhibited, neuronal Ca2+ rhythmic oscillations are impaired, and the SCN loses synchronized rhythms [46]. Specific loss of the Bmal1 gene can delay the phase of autonomous movement in mice by affecting the expression of neuronal clock proteins through the GABA signal [45]. On the other hand, astrocytes can autonomously initiate and maintain the oscillation of SCN clock genes and rhythmic behavioral expression in adult animals and cells. Research has found that transfection of SCN astrocytes with a lentiviral vector overexpressing the Cry gene can restore the daily circadian rhythms and behavior of Cry knockout mice [47]. In addition, disruption of the circadian clock genes also affects the biological functions of astrocytes. Abnormal activation and increased release of pro-inflammatory cytokines can be observed in astrocytes with Bmal1 gene knockout, possibly due to inhibition of the glutathione-S-transferase signaling pathway [48]. The low-affinity nerve growth factor receptor p75NTR is also a circadian clock gene that contains evolutionarily conserved E-box enhancers and can be directly regulated by Clock–Bmal1 heterodimers [49]. It has been found that rhythmic expression of p75NTR can mediate the daily metabolic balance of glucose or glycogen in astrocytes, and lactate generated by glycolysis can be transferred from astrocytes to neurons to meet their energy demands [50].

### 3.4 Circadian cycle and peripheral immune system

In the past, due to the belief that the brain has special anatomical and physiological features, such as the lack of a lymphatic drainage system and specific antigen-presenting cells, as well as the presence of the BBB that restricts the entry of pathogens and immune cells, it has been considered as one of the body’s immune privileged sites. However, current research has shown that there exists a complex lymphatic drainage network in the brain, composed of perivascular spaces (VRS), glial lymphatic system, and meningeal lymphatic vessels, which allows peripheral immune cells to enter the cerebrospinal fluid and VRS under physiological conditions, playing an immunosurveillance role [51,52]. Under pathological stimulation, most cells in the CNS, such as microglia, astrocytes, and neurons, can express the major histocompatibility complex (MHC), playing an antigen-presenting role [53,54]. In addition, sustained activation of microglia and astrocytes can cause BBB leakage, leading to the infiltration, adhesion, and migration of a large number of neutrophils and lymphocytes into the CNS, further mediating neuroinflammatory responses [55]. However, some studies have also shown that in the neuroinflammatory response induced by autoimmune encephalomyelitis models, microglia may not primarily function as antigen-presenting cells, but rather through infiltrated dendritic cells expressing MHC class II molecules to mediate T-cell immune responses [56]. Therefore, there exist complex interactions between the peripheral immune system and the CNS, involving multiple pathological processes in the neuroimmune response.

The circadian cycle plays an important role in the regulation of the peripheral immune system. Most peripheral immune cells, including innate and adaptive immune cells, have their own molecular clock and exhibit significant rhythmic differences during recruitment and activation processes [15,57,58]. Studies have found that the bone marrow chemokine CXCL12 is regulated by the hypothalamic sympathetic–parasympathetic nervous system in a circadian manner, leading to periodic fluctuations in CXCL12 levels and activation of the CXCR4 receptor to maintain the daily rhythmic changes in the number of neutrophils in the blood reserve in the bone marrow [59]. In addition, Bmal1 can regulate neutrophil transcription and migration by controlling the CXCR2 and CXCR4 signaling pathways, respectively, and exert pro-/anti-aging effects on neutrophils [7]. In macrophages, the circadian cycle affects the signaling pathways of macrophage pattern recognition receptors, inflammatory mediators, and phagocytic activity [60]. Krüppel-like factor 4 (KLF4) expression is thought to be a time-specific molecule, and its periodic secretion is involved in the regulation of macrophage phenotype and the rhythmic expression of inflammatory factors. KLF4 expression is downregulated in aging macrophages, and disrupting the circadian rhythms of macrophages after inhibiting KLF4 gene expression further confirms the close relationship between circadian rhythms expression and disruption of the innate immune state [61]. Studies have suggested that the clock gene REV-ERBα mediates the expression of the PI3K/Akt signaling pathway and is involved in regulating the diurnal rhythm of macrophage polarization [62], indicating that REV-ERBα could also be a potential drug intervention target for regulating the circadian rhythms and inflammatory response. Similarly, adaptive immune responses also exhibit rhythmicity. Studies have shown that the strength of the immune response induced by antigen immunization at different times of the day varies significantly. Compared to nighttime, more CD8+ T cells are produced in response to antigen immunization at noon, and the rhythmic response disappears after knocking out the Bmal1 gene in T cells, further confirming the importance of circadian rhythms in regulating adaptive immune responses [63].

## 4 Conclusion

As an important characteristic of adaptive evolution in organisms, the circadian cycle is involved in various life processes at multiple levels, including the organism, organ, and cellular levels. Disruptions in the circadian cycle are closely related to neuroinflammation, where the disruption of the rhythmicity of microglia, astrocytes, and peripheral immune function mediate abnormal phenotypic transformation and secretion activity of inflammatory cells, promoting the development of neuroinflammation. However the mechanisms by which disruptions in the circadian cycle regulate neuroinflammation are not fully understood, and some of which involve the modulation of sleep and the expression of NLRP3 inflammasomes [64,65]. Therefore, further exploration of the molecular patterns of circadian rhythms regulation and the specific mechanisms of its interaction with neuroinflammation may be an important research direction in the future.
